# Left anterior mini-thoracotomy: an alternative approach for pulmonary valve replacement after surgically corrected tetralogy of fallot

**DOI:** 10.1186/s13019-024-02817-5

**Published:** 2024-07-10

**Authors:** Yan Le Ho, Abu Yamin Khamis, Basheer Ahamed Abdul Kareem

**Affiliations:** Department of Cardiothoracic Surgery, Penang General Hospital, George Town, Penang Malaysia

**Keywords:** Minimally invasive valve surgery, Pulmonary valve replacement, Left anterior mini-thoracotomy, Pulmonary regurgitation, Tetralogy of Fallot

## Abstract

**Introduction:**

Pulmonary regurgitation (PR) remains a common sequela in patients following surgically corrected TOF, and may lead to progressive right ventricle dilatation and dysfunction. The conventional approach of redo-sternotomy for pulmonary valve replacement (PVR) is associated with increased operative time as well as risks of bleeding and injury to the heart and great vessels. Thus, left anterior mini-thoracotomy has become an alternative approach in eliminating the risks of redo-sternotomy in these patients. This series aimed to determine the outcomes of minimally invasive pulmonary valve replacement after surgical TOF correction.

**Methods:**

A retrospective analysis was conducted on 24 patients with severe PR post-surgical TOF correction who underwent left anterior mini-thoracotomy PVR in Penang General Hospital from January 2021 to January 2023.

**Results:**

The median age was 23.5 years (I.Q.range 17.6-36.3), with a male:female ratio of 1:4. Majority of patients had mild to moderate symptoms prior to surgery and 19 patients (79.1%) were on regular diuretics medication. All patients had severe free-flow PR with evidence of right ventricular dilatation and dysfunction. Magnetic Resonance Imaging and computed tomography of pulmonary artery were performed prior to surgery. Minimally invasive PVR was performed on all patients via left upper anterior mini-thoracotomy and femoral-femoral bypass without cardioplegic arrest. The operative time and cardiopulmonary bypass time were 208 (I.Q.range 172-324) and 98.6 minutes(I.Q.range 87.4-152.4) respectively. The time to wean off inotropes postoperatively was 6.2 hours (I.Q.range1.4-14.8), and no postoperative arrhythmia and chest re-exploration were reported. Most patients stayed in Intensive Care Unit (ICU) for 10.8 hours (I.Q.range 8.4-36.5), and the total hospital stay was 4.2 days (I.Q.range 3.4-7.6). 2 patients (11.1%) required blood transfusion postoperative. There was no paravalvular leak and no mortality during the follow-up period of up to 28 months.

**Conclusion:**

Minimally invasive PVR after surgical correction of TOF is a safe alternative to the conventional redo-sternotomy approach in patients with favorable anatomy. This approach is able to reduce the risks associated with redo-sternotomy, particularly bleeding and injury to mediastinal structures, with the additional benefit of expedited recovery and hospital discharge. Our series has shown a safe and efficient approach in these patients with favorable outcomes.

## Introduction

Pulmonary regurgitation (PR) is inevitably the sequela of Tetralogy of Fallot (TOF) repair, and the detrimental impact of untreated significant PR on the long-term well-being of these patients is increasingly recognized [1]. PR after TOF repair is generally well tolerated for a long time in most patients. However, chronic significant PR may result in progressive right ventricular dilatation, dysfunction, failure, malignant arrhythmia, and sudden death [2]. Therefore, the necessity of pulmonary valve replacement (PVR) in patients with significant PR after surgical TOF repair is generally accepted [1]. The goal of PVR is to potentially reverse the deleterious hemodynamic effects of significant PR. The standard approach of PVR in these patients has been through repeat median sternotomy. However, this conventional approach of redo-sternotomy has been associated with increased operative time, risk of bleeding, and injury to the heart and great vessels. In patients who have undergone surgical repair of TOF, the presence of transannular patch, dilated right atrium and right ventricle are the structures frequently at risk during dissection in repeat sternotomy [3]. Thus, minimal invasive surgery (MIS) approach by left anterior mini-thoracotomy becomes an alternative approach in eliminating the risk of redo-sternotomy in these patients. The purpose of this study is to determine the results and surgical outcomes of minimally invasive PVR in patients who underwent TOF repair, and to assess the effectiveness and safety of this minimally invasive approach.

## Method

### Patients’ profile

A retrospective study was done on 24 patients who underwent MIS PVR after TOF repair from January 2021 to January 2023 at Department of Cardiothoracic Surgery, Penang General Hospital. Full medical records, including the cardiac database of our institution were reviewed. All patients had more than moderate degree of PR. The severity of PR was assessed by pulse-wave Doppler characteristics of the main pulmonary artery and was graded as moderate or severe if the regurgitant fraction was more than 40%. Right ventricular dysfunction is defined as right ventricular dilatation in the presence of reduced ventricular function as evident by echocardiography and cardiac Magnetic Resonance Imaging (MRI).

### Preoperative evaluation

**Fig. 1:**
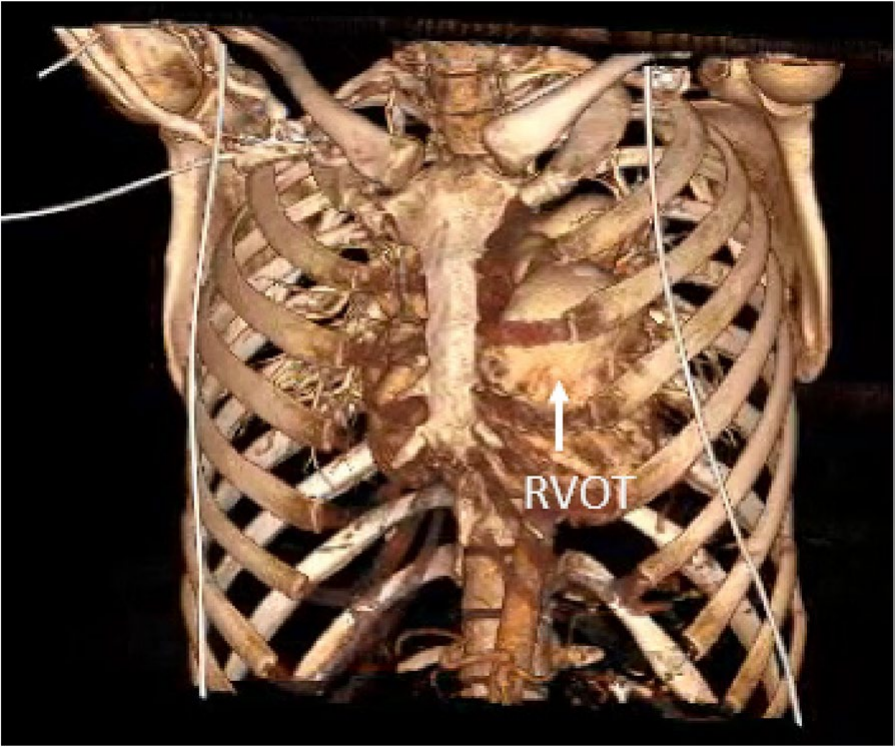
Reconstructed CTPA imaging showing the location of RVOT and main pulmonary artery in relation to the intercostal space and chest wall

The patients who underwent initial TOF repair were followed up by pediatric cardiologists every 6 to 12-month intervals. Careful review of symptoms, physical activities, and signs of right heart failure was performed at every visit. Progressive increase of cardiothoracic ratio in chest X-ray, new onset of symptoms, and decreasing tendency of physical activity were considered indications of diagnostic workups including echocardiography and exercise test. Cardiac MRI was performed to evaluate the severity of PR and RV function objectively, as well as to determine the timing of intervention. Other concomitant intracardiac pathologies such as residue intracardiac shunt or branch pulmonary artery stenosis that requires intervention were identified and excluded. Doppler ultrasound of the groin was performed to evaluate the size and patency of the femoral vessels. A Computed Tomography of Pulmonary Artery (CTPA) was also performed on all patients to delineate the anatomy of the right ventricle, right ventricular outflow tract, and pulmonary arteries. The relationship and relative position of the right ventricular outflow tract (RVOT) and main pulmonary artery (MPA) to the anterior chest wall and intercostal space were also evaluated by CTPA (Fig. 1).

### Indications for PVR

In our practice, the indications for PVR followed the standard guidelines that have been previously published [2]. We consider PVR in (1) symptomatic patients with exercise intolerance and signs of right heart failure, (2) symptomatic arrhythmia, or (3) right ventricular dilatation and dysfunction assessed by 2D echocardiography and MRI. Most patients in the study were symptomatic, and all patients had moderate to severe PR with progressive right ventricular enlargement (median RVEDVI 183.6 ml/m2 (I.Q.range 172.5 – 198.7).

### Operative technique

**Fig. 2:**
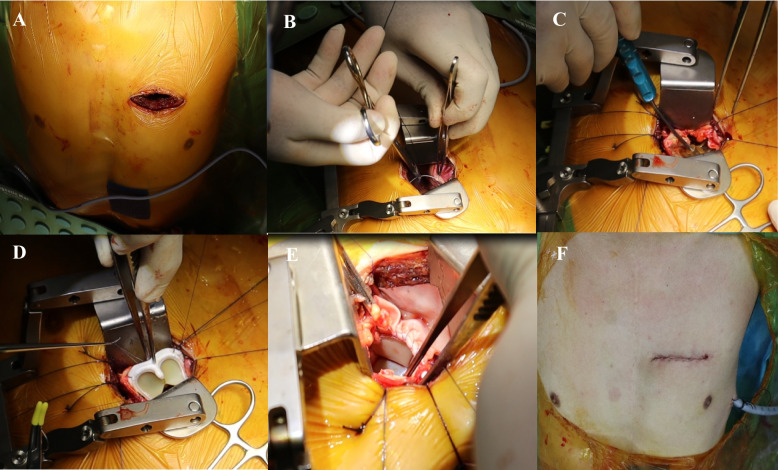
**A** A 5-cm incision is made over the 3rd intercostal space. **B** After adhesiolysis, pericardium is retracted to expose RVOT and main PA. **C** RVOT or the previous transannular patch is opened longitudinally, the annulus was sized appropriately. **D** The prosthesis valve is implanted carefully with running 3/0 polypropylene suture. **E** The completely seated placement of the valve as seen from RVOT, with a patch of bovine pericardium to augment the RVOT and main PA. **F** The final skin incision and a single chest drain is placed

After induction of general endotracheal anesthesia with a single lumen endotracheal tube and placement of adequate monitoring lines, the patient is positioned supine with a sandbag located just underneath the tip of the left scapula. The patient is then prepped and draped from the chin to both knees. We utilize peripheral cannulation to establish cardiopulmonary bypass circulation. A 2-3cm transverse incision is made at the right groin, exposing both the femoral artery and vein. Following administration of heparin systemically, the vessels are cannulated with appropriately sized cannulas. We routinely cannulate the femoral vein followed by femoral artery. Transesophageal echocardiography (TEE) is used to aid the cannulation process to ensure the guidewire and cannula are cannulated in-situ within the great vessels. A 6-cm horizontal incision is made 1cm lateral to the left lateral border of upper sternum at the second or third intercostal space based on the preoperative CTPA findings. The underlying muscle is split bluntly and dissected down until the left pleural cavity is entered. Cardiopulmonary bypass is commenced with the aid of vacuum-assisted drainage and the heart is decompressed. Adhesiolysis is done to separate the pericardium from the left lung, which is then carefully peeled off from the cardiac structures. Pericardial stay sutures are placed accordingly to grant adequate exposure of the right ventricle, infundibulum, and pulmonary artery. The aorta is not cross-clamped. Pulmonary arteriotomy is made with a longitudinal incision and the pulmonary annulus is exposed. The incision can be extended proximally to the RVOT and distally to the pulmonary artery if required. The pulmonary annulus is then sized and an appropriately sized bioprosthetic valve is implanted with running 3/0 Prolene sutures. The pulmonary arteriotomy is either closed directly or with a pyramidal-shaped bovine pericardial patch with a running 4/0 Prolene suture based on the size of bioprosthetic valve. Upon completion, the heart is de-aired and the patient is weaned off cardiopulmonary bypass. Postoperative TEE is done to confirm the satisfactory placement of the pulmonary bioprosthetic valve with no paravalvular leak. Decannulation is done and protamine is administered. A single chest drain is inserted in the left pleural cavity, and the thoracotomy incision is closed in layers in standard fashion. The patient is then transferred to Cardiac Intensive Care Unit and is usually extubated 3 to 4 hours later. Figure 2 shows the stepwise approach of left anterior mini-thoracotomy for PVR.

### Statistical analysis

Baseline characteristics are reported as mean ± SD, median and interquartile range, or ranges for continuous variables, and as counts and percentages for categorical variables. A p-value of less than 0,05 was considered significant.

## Results

### Preoperative profile

Twenty-four patients underwent minimally invasive PVR via left anterior mini-thoracotomy between January 2020 to January 2022. All of these patients had the initial diagnosis of TOF with pulmonary stenosis and 21 patients (87.5%) had transannular patch done, whereas another 3 patients (12.5%) with infundibular stenosis had pulmonary valvulotomy. The median age of PVR was 23.5 years (I.Q. range 17.6 – 36.3), and weight of 39.8 kg (I.Q. range 32.6 – 52.7), with male:female ratio of 1:4. None of the patients required additional catheter interventional or surgical procedures prior to PVR. The pulmonary arteries and RVOT of all patients were within normal range and no residue intracardiac shunt was seen as evident by echocardiography and cardiac MRI. Most of the patients were symptomatic on admission, and 19 patients (79.1%) required anti-failure medications prior to surgery. Echocardiography showed the presence of right ventricular dilatation and dysfunction in all patients, which is also evident in cardiac MRI. Table 1 summarizes the preoperative characteristics and imaging of the patients.

**Table 1:**
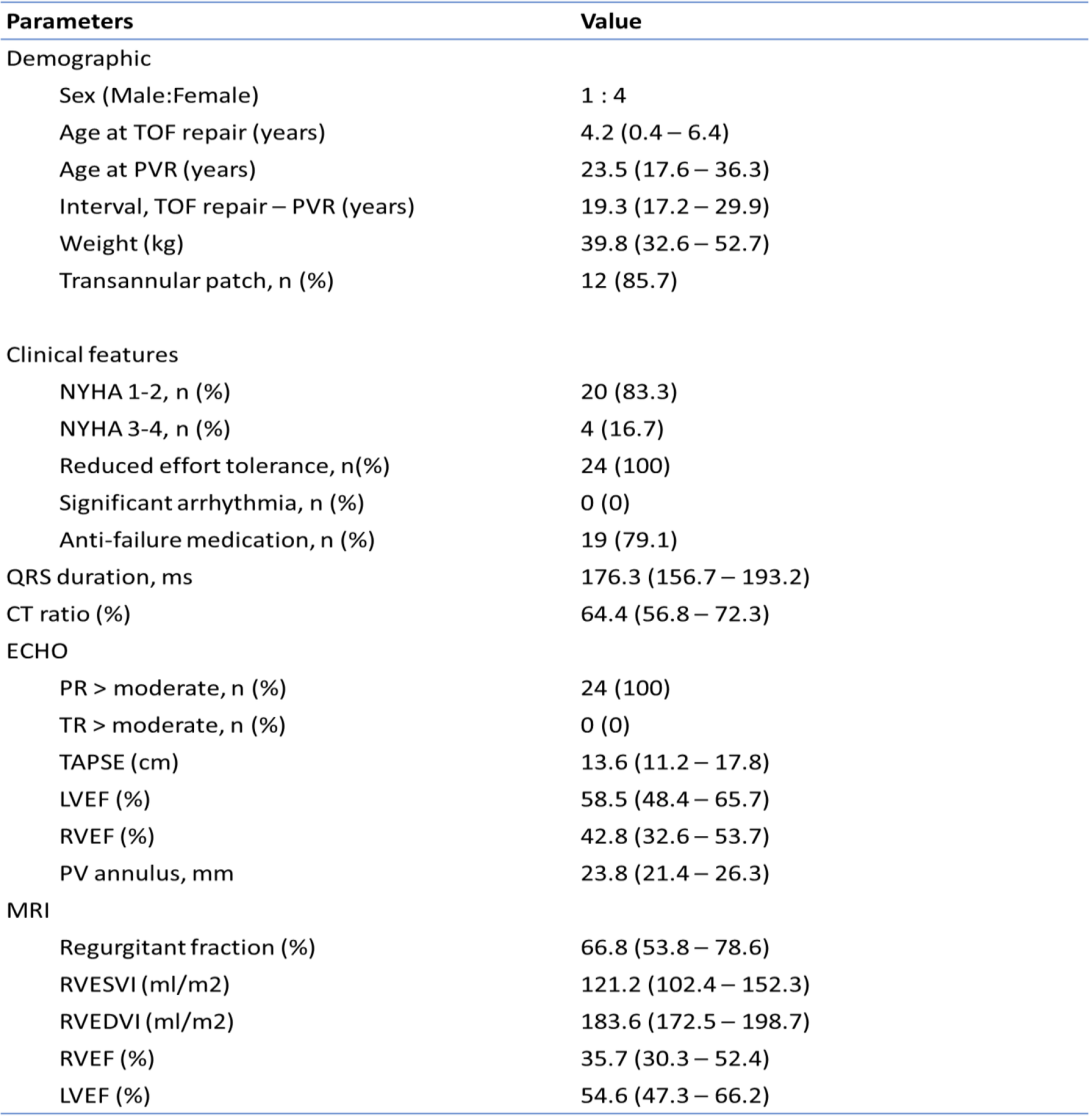
Preoperative characteristics and imaging profile of patients

Values are reported as number (percentage) and median (interquartile range) when appropriate

### Early outcomes

The median cardiopulmonary bypass time was 97.3 minutes (I.Q. range 87.4 – 152.4), with a total operative time of 208 minutes (I.Q. range 172 – 324). Bioprosthetic valve was used in all patients. 21 patients (87.5%) required a bovine pericardial patch to close the anterior rim of the bioprosthesis valve, anterior wall of RVOT, and pulmonary artery. None of the patients required conversion from thoracotomy to sternotomy. Postoperatively, all patients were extubated within 7 hours of surgery, and they were able to transfer out from CICU within 14 hours after surgery. Postoperative echocardiography showed no incidence of paravalvular leak. All patients were able to be discharged home by day 5 of operation. The surgical outcomes are summarized in Table 2.

**Table 2:**
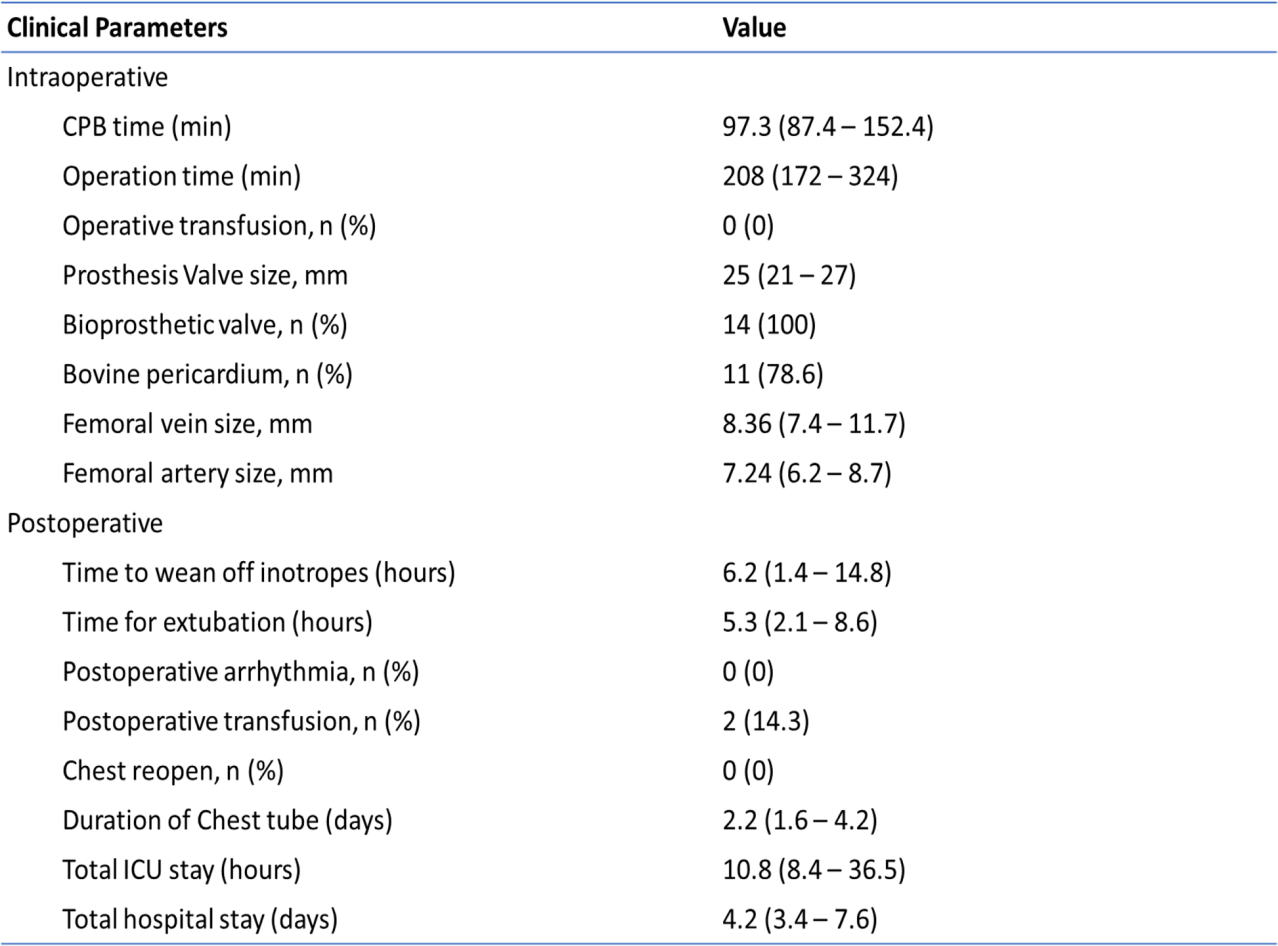
Early surgical outcomes of patients

Values are reported as number (percentage) and median (interquartile range) when appropriate

Two patients developed persistent high-output drainage from the left pleural cavity, but both chest drains were removed on day 4 after surgery with no subsequent pleural collection. No incidence of empyema and diaphragm paresis was reported. There was also no reported case of prosthesis valve endocarditis. No in-hospital and 30-day mortality was reported. Table 3 summarizes the early postoperative complications of our patients.

**Table 3:**
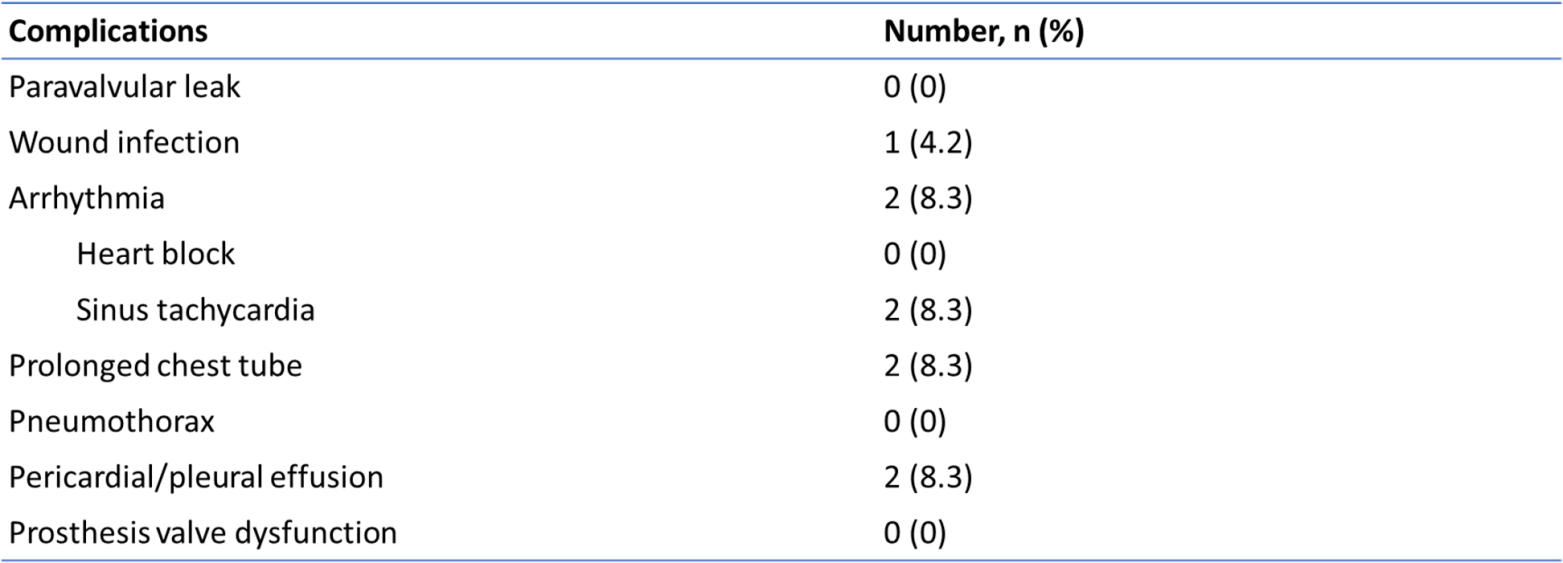
Postoperative complications

### Late outcomes

The maximum follow-up period was up to 28 months. There was a significant improvement in the functional status of the patients after PVR (p<0.028). The cardiothoracic ratio and right ventricular function also showed a significant improvement after PVR. The comparison of preoperative and postoperative clinical profiles is summarized in Table 4. The freedom for reintervention was 100%. None of the patients required hospital readmission and there was no late mortality reported.

**Table 4:**
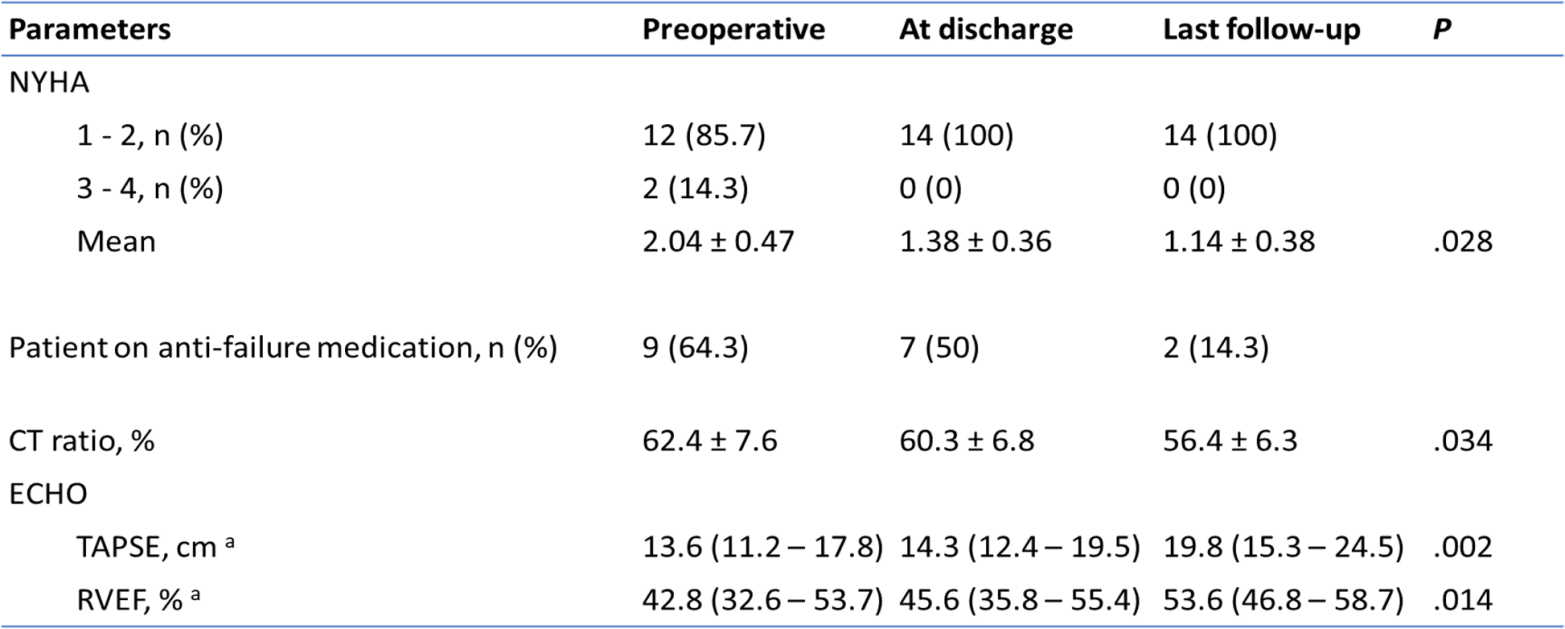
Comparison of preoperative and postoperative clinical profiles

^a^ Values are reported as median (interquartile range)

## Discussion

Surgical repair of TOF with pulmonary stenosis has improved dramatically over the past few decades with low mortality and morbidity. Often the integrity of the stenosed pulmonary valve is disrupted to effectively relieve RVOT obstruction, which results in pulmonary regurgitation that eventually worsens over time. The ensuing chronic right ventricular dilatation caused by volume overload leads to akinetic and dyskinetic of the right ventricular wall, which consequentially results in the initiation of a series of pathophysiological abnormalities and hemodynamical disturbances. This process ultimately results in right ventricular dysfunction and failure as seen in all of our patients [4]. Indeed, the presence and degree of pulmonary regurgitation influence exercise tolerance and incidence of atrial and ventricular arrhythmias, and the risk for sudden unexpected cardiac death. Therefore, PVR is indicated as the adverse remodeling manifests in increasing rates of morbidity and mortality beginning during the third decade of life [5]. Early PVR in this group of patients has been shown to improve ventricular function and functional class, stabilize QRS duration, and reduce atrial and ventricular arrhythmias [5, 6].

Minimally invasive cardiac surgery has become a routine approach for primary mitral, tricuspid, and even aortic valve surgery. In recent years, the benefit of a separate smaller incision in minimally invasive cardiac surgery away from the previous sternotomy site has gained popularity among cardiac surgeons, owning to the advantage of reduced tissue dissection and thus decreased incidence of injury to mediastinal and cardiac structures. Filip et al reported their findings in 80 patients who underwent redo mitral valve surgery using minimally invasive thoracotomy and demonstrated the safety of this approach in reoperations [7]. Murzi et al have successfully undergone reoperative minimal invasive mitral valve surgery in 173 patients with good early and late outcomes [8]. Based on our experience, we recognized the advantages of minimally invasive surgery for reoperative procedures, and extended this technique to patients who required repeat sternotomy. Left anterior mini-thoracotomy has emerged as a possible alternative for PVR in patients who have undergone TOF repair. However, the use of minimally invasive technique for PVR in redo operations is not well described and reported, and the literature is only limited to a few case reports and series. Cheema et al have successfully performed a PVR through left thoracotomy in a patient with PV endocarditis, whereas Nellis has successfully resected a pulmonary fibroelastoma via the minimally invasive approach [9, 10]. Our current findings confirmed the surgical feasibility and outcomes of these previously published reports. In our opinion, the potential benefits of this approach are: avoidance of sternal reentry, limited substernal and cardiac dissection, early recovery and mobilization, decreased sternal wound complications, decreased overall mortality, and decreased length of hospital stay. Furthermore, the avoidance of repeat sternotomy in these patients prevents sternal trauma and lessens mediastinal dissection, resulting in lesser blood transfusion and transfusion-related morbidity [7, 8].

Minimally invasive PVR through a left anterior mini-thoracotomy provides good exposure to RVOT, main pulmonary artery, and branch pulmonary artery. It allows satisfactory placement of an adequate-sized pulmonary valve prosthesis in the same way as it is done through a sternotomy, and we have proved that it can be successfully performed in redo operations. This is also supported by the fact that in our series, no patient required conversion to sternotomy because of difficult pulmonary valve exposure or major vascular injury. Moreover, this minimally invasive approach guarantees a safe and excellent view of the RVOT and pulmonary artery. Preoperative CTPA is an excellent imaging modality and the 3D reconstruction is valuable in providing an adequate insight into the surgical approach. The second or third left intercostal provides direct and straight access to the RVOT and main pulmonary artery without requiring extensive cardiac mobilization as in standard redo procedures through repeat sternotomy. Based on our experience, these areas are not as adherent to the surrounding structures such as the pericardium, left lung parenchymal, and chest wall, as compared to redo midline sternotomy. The adhesion was in fact, minimal and easily dissected from these structures.

A prerequisite to applying this minimally invasive PVR technique is adequate vascular access for peripheral CPB cannulation. Successful femoral cannulation is crucial to ensure satisfactory CPB bypass during minimally invasive cardiac surgery. In our cohort, preoperative femoral Doppler ultrasonography was done to assess the size and patency of groin vessels thoroughly. Doppler ultrasound also enables the detection of vascular disease, the extent of which will determine the feasibility of femoral cannulation and avoid vascular complications during cannulation. Most of our patients in the series were less than 25 years of age, with a median age of 23.5 years old. Due to the relatively small size of groin vessels in our patients, we routinely perform limited cutdown to identify and expose both the femoral artery and vein. We limit the dissection to the anterior vessel wall and avoid excessive manipulation around the artery to prevent spasm and bleeding. We also successfully avoided vascular-related complications such as femoral artery pseudoaneurysm and dissection by cannulating under direct vision, with the aid of transesophageal ultrasonography to ensure the guidewire and cannula are properly cannulated in-situ within the great vessels, and securing hemostasis at the cannulation site after decannulation. Based on our experience, vacuum-assisted drainage represents a major improvement in cardiopulmonary bypass, allowing adequate venous drainage through a smaller cannula as the femoral vessels in these patients are usually small. We were able to achieve full cardiopulmonary bypass circulation with this technique without the need for neck vessel cannulation.

One of the principal concerns involving the minimally invasive approach in cardiac surgery is the ‘trade-off’ of surgical incision and limited exposure versus the safety of the established approach through full sternotomy [11]. However, if repeated median sternotomy is performed where dense substernal and pericardial adhesion is expected, an incision away from the previous incision site may well be safer as it reduces the risk of injury to the cardiac structure and great vessels. Furthermore, with more experience gained and shorter CPB time, this minimally invasive approach might benefit the patients more than conventional redo sternotomy due to fewer pulmonary and cerebral complications. In our cohort, our experience on CPB with left anterior mini-thoracotomy PVR was only 97.3 minutes (I.Q.range 87.4 - 152.4) with a total operative time of 208 minutes (I.Q.range 172 – 324), which was generally shorter compared to those who underwent repeat sternotomy. Although only right-sided chambers were open during the procedure, the use of CO^2^ insufflation during the surgery could further minimize the concerns related to de-airing and the resultant risk of stroke.

Owning to less tissue dissection and reduced surgical trauma in minimally invasive PVR, patients are expected to have earlier recovery and a shorter course of in-hospital stay postoperatively. Indeed, rapid postoperative recovery is a crucial advantage of minimally invasive surgery, including shorter ICU and overall hospital stay [4, 5, 11, 12]. This trend is consistent with our cohort of patients where all of them were discharged by day 4 of surgery, with 4 patients discharged on day 3 of surgery. All patients were able to be extubated within 7 hours of surgery. With shorter operative time and lesser postoperative pain compared to repeat sternotomy, our patients received their first postoperative physiotherapy treatment in CICU two to three hours after extubation with passive mobilization in the bed, respiratory exercises, and active mobilization to the upright position beside the bed. On the first postoperative day, patients walked with physiotherapist support on the floor. Half of the patients were able to ambulate 20 meters unassisted on the first operative day. On the second postoperative day, the patients were encouraged to exercise independently and half of them were able to ambulate 50 meters unassisted. Postoperative Barthel index was used as an ordinal scale to measure objectively the performance in 10 activities of daily living (ADL) including feeding, personal toilet use, bathing, dressing and undressing, getting on and off the toilet, bladder control, bowel control, moving from a wheelchair to a bed and back, walking on a level surface (or propelling a wheelchair if unable to walk), and ascending and descending stairs. The mean Barthel index for our patient was 44.7 on postoperative day 1, increasing to 92.3 prior to discharge compared to 75.3 reported by Han et al in patients under usual postoperative care after CABG [13]. We believe that avoiding repeat sternotomy, minimizing tissue dissection, and shorter CPB time were the key contributors to the earlier commencement of independent ambulation, with the added benefits of improved respiratory function, increased mobility, and reduced risk of surgical site infection as well as pneumonia and hospital stay. Our findings are consistent with a meta-analysis review by Haya et al showing a similar trend in the recovery of patients after minimally invasive cardiac surgery [14]. In our practice, patients undergoing minimally invasive PVR are advised to have home rest for 2 weeks without any activity restriction, whereas patients receiving median sternotomy require sternal precautions which limits their activity for 6 weeks. We think this is particularly important in the context of paediatric and congenital cardiac surgery as these patients tend to be younger, more active, and often require many subsequent reoperations.

Although we did not encounter any patients who required conversion from thoracotomy to sternotomy in this series, we considered patients with unfavorable anatomy such as dextrocardia, pectus deformity, and body mass index of more than 40kg/m^2^ as relative contraindications, and we counseled as such with the risk of conversion to sternotomy. Furthermore, patients who require concomitant cardiac procedures such as closure of residue intracardiac shunt or other valvular interventions are contraindicated for this minimally invasive approach. In our experience, 4 patients were unable to proceed with this approach: 1 patient with severe pectus deformity as the PA was situated retrosternally with the concern of proper valve placement, 2 patients with moderate-severe tricuspid regurgitation, and 1 patient with residue intracardiac shunt. We did not compare the minimally invasive approach with our standard sternotomy group due to the small sample size in this series. However, we believe left anterior mini-thoracotomy is able to offer a safe alternative to these patients, as well as reduce the length of hospital stay, provide faster recovery, and return to normal daily activities.

## Conclusion

PVR via left anterior mini-thoracotomy after surgical correction of TOF is a safe and effective alternative strategy to the conventional redo-sternotomy approach in patients with favorable anatomy. This approach allows good surgical results in terms of operating time, prosthesis sizing, patient selection, and clinical outcomes. This minimally invasive approach is also able to reduce the risks associated with redo-sternotomy, particularly bleeding and injury to mediastinal structures, with the additional advantage of expedited recovery and hospital discharge. Our series has shown a safe and efficient approach in these patients with favorable outcomes. Longer-term data with a larger number of patients will be needed to provide more insights and allow comparison of outcomes with the standard sternotomy group.

## Data Availability

No datasets were generated or analysed during the current study.

## Abbreviations

CPB: Cardiopulmonary bypass
CTPA: Computed tomography of pulmonary artery
MPA: Main pulmonary artery
PR: Pulmonary regurgitation
PVR: Pulmonary valve replacement
RVOT: Right ventricular outflow tract
TEE: Transesophageal echocardiography
TOF: Tetralogy of Fallot
